# Machine Learning for Predicting Risk and Prognosis of Acute Kidney Disease in Critically Ill Elderly Patients During Hospitalization: Internet-Based and Interpretable Model Study

**DOI:** 10.2196/51354

**Published:** 2024-05-01

**Authors:** Mingxia Li, Shuzhe Han, Fang Liang, Chenghuan Hu, Buyao Zhang, Qinlan Hou, Shuangping Zhao

**Affiliations:** 1 Department of Critical Care Medicine Xiangya Hospital of Central South University Changsha China; 2 Department of Critical Care Medicine ZhuJiang Hospital of Southern Medical University Guangzhou China; 3 Department of Obstetrics and Gynecology, 967th Hospital of the Joint Logistics Support Force of the Chinese People's Liberation Army Dalian China; 4 Department of Hematology and Critical Care Medicine The Third Xiangya Hospital Central South University Changsha China; 5 National Clinical Research Center for Geriatric Disorders Changsha China; 6 Hunan Provincial Clinical Research Center of Intensive Care Medicine Changsha China

**Keywords:** acute kidney disease, AKD, machine learning, critically ill patients, elderly patients, Shapley additive explanation, SHAP

## Abstract

**Background:**

Acute kidney disease (AKD) affects more than half of critically ill elderly patients with acute kidney injury (AKI), which leads to worse short-term outcomes.

**Objective:**

We aimed to establish 2 machine learning models to predict the risk and prognosis of AKD in the elderly and to deploy the models as online apps.

**Methods:**

Data on elderly patients with AKI (n=3542) and AKD (n=2661) from the Medical Information Mart for Intensive Care IV (MIMIC-IV) database were used to develop 2 models for predicting the AKD risk and in-hospital mortality, respectively. Data collected from Xiangya Hospital of Central South University were for external validation. A bootstrap method was used for internal validation to obtain relatively stable results. We extracted the indicators within 24 hours of the first diagnosis of AKI and the fluctuation range of some indicators, namely delta (day 3 after AKI minus day 1), as features. Six machine learning algorithms were used for modeling; the area under the receiver operating characteristic curve (AUROC), decision curve analysis, and calibration curve for evaluating; Shapley additive explanation (SHAP) analysis for visually interpreting; and the Heroku platform for deploying the best-performing models as web-based apps.

**Results:**

For the model of predicting the risk of AKD in elderly patients with AKI during hospitalization, the Light Gradient Boosting Machine (LightGBM) showed the best overall performance in the training (AUROC=0.844, 95% CI 0.831-0.857), internal validation (AUROC=0.853, 95% CI 0.841-0.865), and external (AUROC=0.755, 95% CI 0.699–0.811) cohorts. In addition, LightGBM performed well for the AKD prognostic prediction in the training (AUROC=0.861, 95% CI 0.843-0.878), internal validation (AUROC=0.868, 95% CI 0.851-0.885), and external (AUROC=0.746, 95% CI 0.673-0.820) cohorts. The models deployed as online prediction apps allowed users to predict and provide feedback to submit new data for model iteration. In the importance ranking and correlation visualization of the model’s top 10 influencing factors conducted based on the SHAP value, partial dependence plots revealed the optimal cutoff of some interventionable indicators. The top 5 factors predicting the risk of AKD were creatinine on day 3, sepsis, delta blood urea nitrogen (BUN), diastolic blood pressure (DBP), and heart rate, while the top 5 factors determining in-hospital mortality were age, BUN on day 1, vasopressor use, BUN on day 3, and partial pressure of carbon dioxide (PaCO_2_).

**Conclusions:**

We developed and validated 2 online apps for predicting the risk of AKD and its prognostic mortality in elderly patients, respectively. The top 10 factors that influenced the AKD risk and mortality during hospitalization were identified and explained visually, which might provide useful applications for intelligent management and suggestions for future prospective research.

## Introduction

Acute kidney injury (AKI), as a common complex heterogeneous syndrome in critically ill patients, is associated with an increased risk of death and adverse renal events [1-3]. AKI is more common in elderly patients in the intensive care unit (ICU), with sustained impaired renal function associated with a poor prognosis for survival [4,5]. Therefore, in this study, we focused on AKI in the elderly with renal function impairment for more than 7 days during hospitalization, that is, acute kidney disease (AKD). In 2012, Kidney Disease: Improving Global Outcomes (KDIGO) first proposed the term “AKD,” but at this time, it was viewed as a period of kidney pathology following AKI, not as an independent definition [6]. In a comparative study on the epidemiology of AKD, patients with AKI who developed AKD had a higher risk of chronic kidney disease (CKD) and end-stage renal disease (ESRD), suggesting the potential clinical research value for AKD as a novel term [7]. In 2017, the Acute Disease Quality Initiative (ADQI) 16 Workgroup presented an expert consensus on AKD, defining AKD as AKI with KDIGO stage 1 or higher within 7-90 days of the first diagnosis of AKI [8]. There is a distinct disease course between AKI, AKD, and CKD. AKD highlights the key intervenable period in the transformation process from AKI to CKD and lays the foundation for the construction of management strategies for renal function recovery.

Recently, a multicenter study indicated that more than half of the hospitalized patients with AKI developed AKD, which increased the risk of long-term mortality [9]. Nevertheless, an epidemiological study by Andonovic et al [10] found that patients with AKD in the ICU have a higher short-term risk of death but no statistically significant difference in long-term survival. Moreover, Chen et al [11] reported that patients with AKD are more likely to require long-term dialysis. Considering the high incidence and mortality rate of AKD, researchers have conducted exploratory early warning studies on AKD. Current predictive research on AKD has focused primarily on patients with AKI during hospitalization, sepsis-related AKI, coronary heart disease, and renal cell carcinoma postoperatively [9,12-14]. However, it should be noted that elderly patients with diminished renal function have received insufficient attention, and little is known about the AKD risk and prognostic mortality in the elderly. Further, some studies have explored the use of artificial intelligence (AI) algorithms to predict the onset and progression of diseases, but only a few have developed user-friendly online prediction apps for clinical practice. Zhou et al [15] established an online calculator using Extreme Gradient Boosting (XGBoost) to predict AKI in patients with sepsis-associated acute respiratory distress syndrome. Regrettably, this risk calculator has not been validated externally to determine generalization capacity. Thus, it is imperative to use big data and AI technology to conduct research on the diagnosis and prognosis prediction of AKD in the elderly, and transform the AI models into internet-based apps to assist clinicians in timely intervention to maximize the improvement in renal function and survival outcomes.

Therefore, this study intended to develop 2 machine learning prediction models: one was a model for predicting the risk of AKD in critically ill patients during hospitalization to address problems regarding renal function recovery and early detection of AKD; the other was for predicting the in-hospital mortality in AKD to deal with the adverse outcomes of AKD. In addition, Shapley additive explanation (SHAP) analysis was used to rank the importance and visualize the correlation of the features affecting the occurrence and outcome of AKD [16]. Importantly, we deployed the models with the most comprehensive performance as web-based prediction apps to facilitate doctors' decision-making.

## Methods

### Ethical Considerations

This study was conducted strictly in accordance with the Guidelines for Developing and Reporting Machine Learning Predictive Models in Biomedical Research [17]. The MIMIC-IV database was approved by the Ethics Review Boards of the MIT and the Beth Israel Deaconess Medical Center. This study obtained access and download permission from the MIMIC-IV database (no. 41817305) and passed the retrospective ethics review of the Medical Ethics Committee of Xiangya Hospital Central South University (no. 202105200). Due to the deprivacy of the data for this retrospective study, it was exempted from patients’ informed consent.

### Study Design

A retrospective cohort study was conducted using electronic health records (EHRs) from the Medical Information Mart for Intensive Care IV (MIMIC-IV) data and patients’ data from Xiangya Hospital Central South University (Hunan, China) [18]. In June 2022, the Massachusetts Institute of Technology (MIT) released the revised version of MIMIC-IV 2.0, which contained in-hospital diagnosis and treatment records, as well as in- and out-of-hospital death information for about 40,000 patients in ICUs, and achieved data privacy by deleting patient identification numbers and drifting data through time. The MIMIC-IV cohorts were used to develop machine learning predictive models. Further, the Department of Critical Care Medicine at Xiangya Hospital Central South University is the National Key Clinical Specialty. Approximately 2500 critically ill patients are admitted to the department each year for the treatment of various diseases. Patients were enrolled in the Department of Critical Care Medicine from 2017 to 2021 for external validation of models.

### Data on AKI in the Elderly

#### Inclusion and Exclusion Criteria

**Figure 1:**
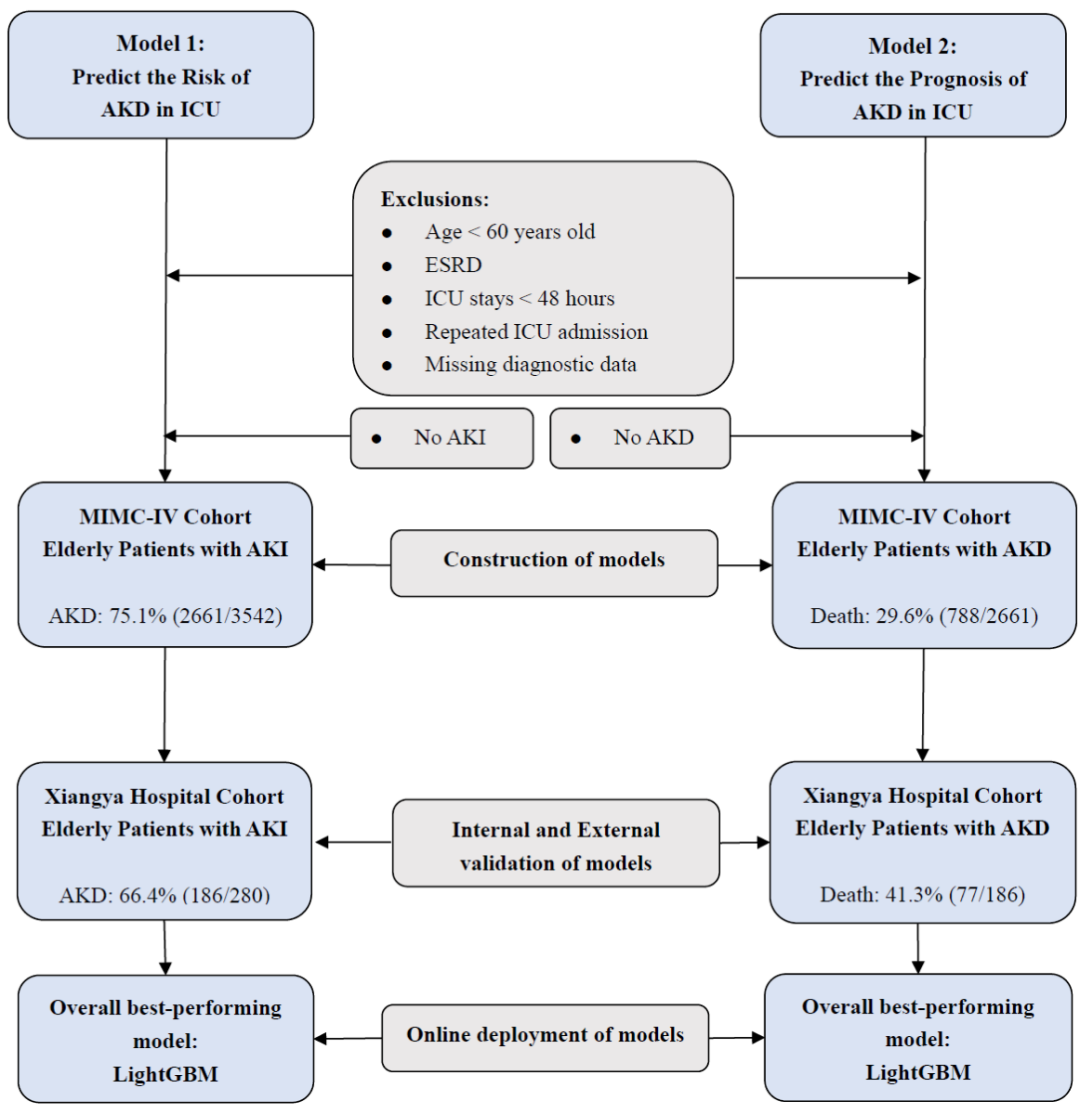
Flowchart for the selection of elderly patients with AKI and AKD. AKD: acute kidney disease; AKI: acute kidney injury; ESRD: end-stage renal disease; ICU: intensive care unit; LightGBM: Light Gradient Boosting Machine; MIMIC-IV: Medical Information Mart for Intensive Care IV.

Data on AKI in elderly patients in the ICU were collected for the construction and external verification of an early warning model for the risk of AKD. According to the Chinese Healthy Elderly Standard issued by the National Health Commission of China, those 60 years old and above were defined as the elderly. The inclusion criteria were (1) age≥60 years, (2) ICU stay of at least 48 hours, (3) EHRs of patients admitted to the ICU for the first time, and (4) patients with AKI who met KDIGO criteria. The exclusion criteria were (1) patients with ESRD and (2) missing data on the diagnosis of AKI. Figure 1 shows the data extraction process in detail.

#### Outcome Definition

The occurrence of AKD during hospitalization was considered the outcome of the risk prediction study. AKI was diagnosed and staged in accordance with the AKI guidelines issued by the KDIGO in 2012 [6]. According to the expert consensus of the ADQI-16 Workgroup in 2017, AKD was defined as the presence of at least stage 1 AKI within 7-90 days after the initial diagnosis of AKI [8]. In this study, patients with AKI who met this definition during hospitalization were regarded as having AKD (Multimedia Appendix 1).

#### Data Extraction

Navicat Premium (version 15.0.13) was used for MIMIC-IV database management and PostgreSQL (version 9.6; PostgreSQL Global Development Group) for variable extraction. Patients with AKI were identified based on their serum creatinine and urine output levels. Patients with AKI stage higher than or equal to 1 between 7 days following AKI and discharge were considered to have AKD during their hospitalization. Finally, 33 variables were determined and extracted from the Xiangya Hospital data set and the MIMIC-IV database, including age, gender, and the AKI stage as basic characteristics; sepsis, hypertension, diabetes, chronic kidney disease (CKD), chronic pulmonary disease (CPD), and chronic liver disease (CLD) as comorbidities; mechanical ventilation (MV), renal replacement therapy (RRT), and vasopressors as interventions; heart rate, respiratory rate, systolic blood pressure (SBP), diastolic blood pressure (DBP) as vital signs; and white blood cell (WBC) count, red blood cell (RBC) count, hemoglobin, hematocrit, potassium, calcium, anion gap, partial pressure of oxygen (PaO_2_), partial pressure of carbon dioxide (PaCO_2_), pH, glucose, blood urea nitrogen (BUN), and serum creatinine as laboratory tests. These examination indicators were measured on day 1 of AKI diagnosis. We also obtained BUN and serum creatinine levels on day 3 following AKI diagnosis, as well as corresponding delta BUN and delta creatinine values on day 3 minus day 1.

### Data on AKD in the Elderly

#### Inclusion and Exclusion Criteria

Following the aforementioned study on AKI in the elderly, further extracted the data concerning critically ill patients with AKD to construct and verify a model that predicted poor prognostic mortality during hospitalization. The inclusion criteria of elderly patients with AKD were as follows: (1) age≥60 years, (2) length of stay in the ICU for more than 48 hours, (3) patients admitted to the ICU for the first time, and (4) patients with AKD who met the ADQI consensus of 2017. The exclusion criteria were (1) patients who had ESRD and (2) missing data related to AKD diagnosis. A detailed description of the data extraction can be seen in Figure 1.

#### Outcome Definition and Data Extraction

In-hospital death was the outcome of the prognostic prediction study of AKD in the elderly.

This study on prognostic mortality prediction and the aforementioned study on risk prediction of AKD in the elderly were similar in terms of the content and timing for extracting 33 variables.

### Construction and Validation of Models

Several supervised learning algorithms were selected to solve classification prediction problems in this study: logistic regression model (LRM), XGBoost, Light Gradient Boosting Machine (LightGBM), multilayer perceptron (MLP), random forest (RF), and the K-nearest neighbor (KNN) algorithm. Two models were developed using the MIMIC-IV cohort: one for predicting AKD occurrence among the elderly and the other for predicting the prognostic mortality in AKD. To prevent overfitting and improve generalization, a 10-fold cross-validation method was applied to assess the models, and the final models were constructed based on repeated iterations. Multimedia Appendix 2 shows the optimal hyperparameters of the AKD risk and AKD mortality models. Through the GridSearchCV module, we conducted a grid search that traversed all parameter values and returned the parameter combination that provided the best overall performance. The models constructed from the MIMIC-IV database were internally validated by bootstrap resampling with replacement to evaluate performance. The established training model was externally validated with the Xiangya Hospital cohort.

### Evaluation and Deployment of Models

The classification prediction effect of the models was evaluated using the area under the receiver operating characteristic curve (AUROC), sensitivity, specificity, positive predictive value (PPV), and negative predictive value (NPV) under the optimal cutoff value. Moreover, a calibration curve was developed to determine the models’ predictive accuracy, and clinical decision curve analysis (DCA) was performed to assess their clinical utility. To enhance the interpretability of machine learning black-box models, we performed SHAP analysis by visualizing each feature’s marginal contribution to the models’ prediction in importance-ranking plots and showing how each feature impacts the outcome in partial dependence plots. Lastly, we selected machine learning algorithm models with the best comprehensive performance from the training and validation cohorts and deployed them to the online server for the convenience of clinical workers or patients. The web-based apps were managed by Heroku.

### Statistical Analysis

Data were analyzed using Python (version 3.9.7) and R (version 4.2.0; R Foundation for Statistical Computing). Variables with a missing ratio higher than 35% were deleted, and the *mice* package (version 3.14.0) in R was used to fill in the missing values using the multiple imputation method. In data preprocessing, the Z-score method was used to scale the continuous variables with the *StandardScaler* function. Categorical variables were represented as numbers (percentages) and compared between groups using the chi-square test. Depending on whether the continuous variables were normally distributed, the mean (SD) or median (IQR) was expressed and compared using the 2-tailed *t* test or the Mann-Whitney *U* test. By analyzing the Youden index, the optimal cutoff value of the receiver operating characteristic (ROC) curve was calculated, as well as the sensitivity, specificity, PPV, and NPV of the models. Statistical significance was set at *P*<.05.

## Results

### Model 1: Predicting the risk of AKD in the Elderly

#### Baseline Characteristics

In the study on the risk prediction of AKD in critically ill elderly patients during hospitalization, a total of 3542 elderly patients with AKI from the MIMIC-IV database and 280 from Xiangya Hospital were retrospectively included after screening by the inclusion and exclusion criteria as the training and external validation cohorts, respectively. AKD incidence was 75.1% (2661/3542) in the training cohort and 66.4% (186/280) in the external validation cohort. A comparison of baseline characteristics and stratification of the 2 cohorts according to the presence or absence of AKD is shown in Multimedia Appendix 3. In the MIMIC-IV cohort, patients with AKD had a higher proportion of comorbidities (sepsis, diabetes, CKD, and CPD) and a lower proportion of hypertension (*P*<.05); a higher proportion of interventions (MV, RRT, and vasopressor use; *P*<.05); a higher heart rate and lower SBP and DBP in terms of vital signs (*P*<.05); and higher potassium, anion gap, BUN on day 1 (following AKI diagnosis), serum creatinine on day 1, BUN on day 3 (following AKI diagnosis), and serum creatinine on day 3 and lower PaO_2_ in terms of laboratory tests (*P*<.05). Furthermore, more patients with AKD in the Xiangya Hospital cohort were males and had stage 3 AKI (*P*<.05). The features of the Xiangya Hospital cohort with a similar trend to the MIMIC-IV cohort were as follows: diabetes, CKD, RRT, vasopressor use, potassium, anion gap, PaO_2_, BUN on day 1, serum creatinine on day 1, BUN on day 3, and serum creatinine on day 3, while hypertension and SBP had opposite trends and statistical results (*P*<.05).

#### Model Comparison

**Figure 2:**
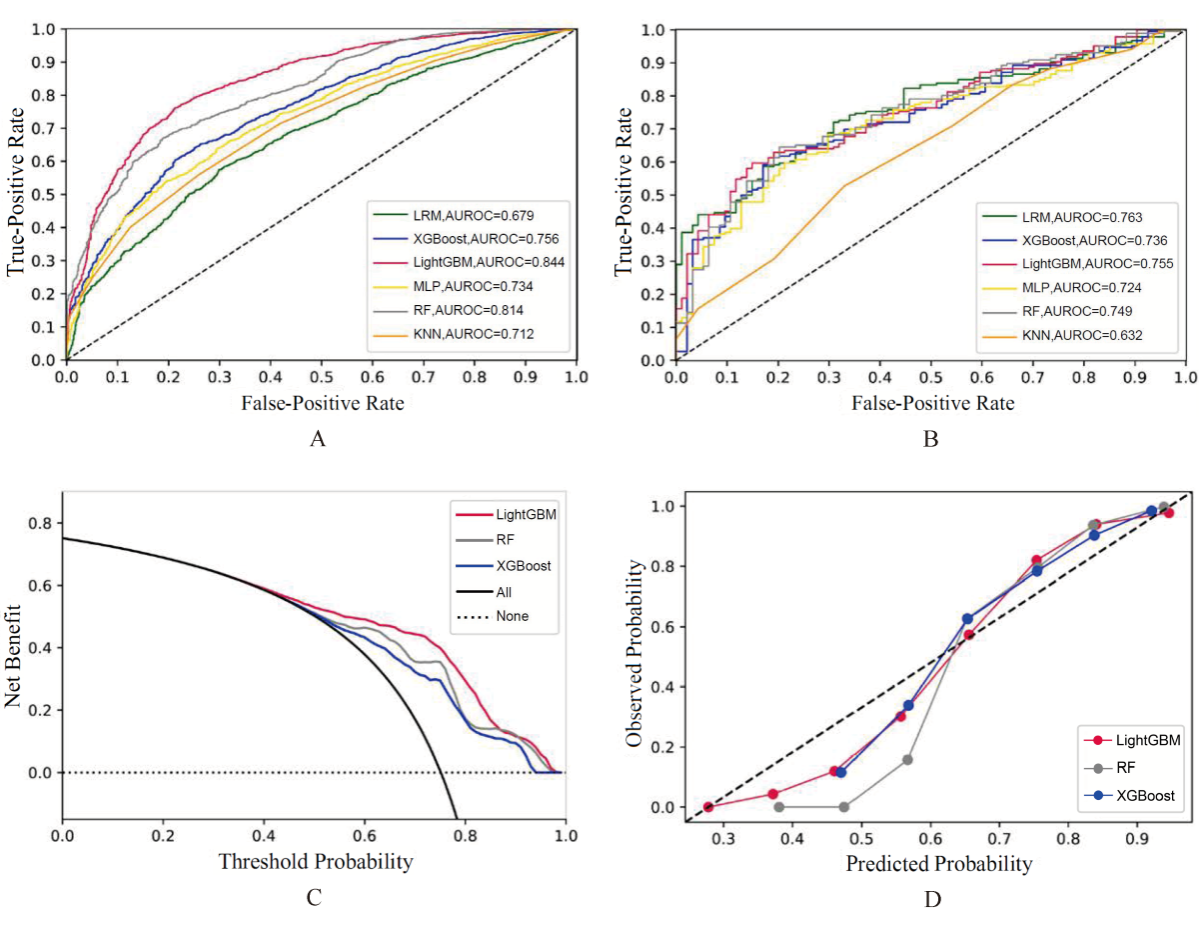
ROC curves, DCA , and calibration curves of AKD risk prediction models. AKD: acute kidney disease; AUROC: area under the receiver operating characteristic curve; DCA: decision curve analysis; KNN: K-nearest neighbor; LightGBM: Light Gradient Boosting Machine; LRM: logistic regression model; MLP: multilayer perceptron; RF: random forest; ROC: receiver operating characteristic; XGBoost: Extreme Gradient Boosting.

We included all the variables shown in Multimedia Appendix 3 in the model construction since these indicators are common and easily obtainable in clinical practice. Table 1 shows the performance comparison of the 6 machine learning models for predicting AKD risk in the training, internal validation, and external cohorts. In the training cohort, the algorithm with the greatest performance was LightGBM, with AUROC=0.844 (95% CI 0.831-0.857), sensitivity=0.788 (95% CI 0.759-0.814), specificity=0.761 (95% CI 0.745-0.777), PPV=0.522 (95% CI 0.495-0.549), and NPV=0.915 (95% CI 0.903-0.927). In the external validation cohort, the best-predicting model was the LRM, which had AUROC=0.763 (95% CI 0.707-0.818), sensitivity=0.830 (95% CI 0.738-0.899), specificity=0.586 (95% CI 0.512-0.658), PPV=0.503 (95% CI 0.422-0.584), and NPV=0.872 (95% CI 0.800-0.925). LightGBM also demonstrated the ability to distinguish patients at a higher risk of AKD during hospitalization in the validation cohort: AUROC=0.853 (95% CI 0.841-0.865), sensitivity=0.817 (95% CI 0.791-0.842), specificity=0.759 (95% CI 0.742-0.775), PPV=0.534 (95% CI 0.507-0.560), and NPV=0.925 (95% CI 0.913-0.936) in the internal validation cohort and AUROC=0.755 (95% CI 0.699-0.811), sensitivity=0.851 (95% CI 0.763-0.916), specificity=0.597 (95% CI 0.523-0.668), PPV=0.516 (95% CI 0.435-0.597), and NPV=0.888 (95% CI 0.819-0.937) in the external cohort. Figures 2A and 2B provide the ROC curves of the prediction models in the training and external cohorts, among which LightGBM showed the best overall performance. Multimedia Appendix 4 exhibits the ROC curves for the internal validation cohort. We selected the 3 algorithms (LightGBM, RF, XGBoost) with better performance in the validation cohort to conduct DCA; Figure 2C shows that when the threshold probability of AKD reached 60%, the net benefit ratio of taking intervention measures was 0.5, showing good clinical applicability of LightGBM. Further, the calibration curves for the 3 algorithms are presented in Figure 2D displaying the relative consistency between predictions and actual values. However, according to Figure 2D, when the threshold probability was low, the prediction probability of the model was high with overfitting, which was consistent with Figure 2C: when the threshold probability was low, the net benefit of the model hardly increased.

**Table 1:** Performance of the AKD^a^ risk prediction models for elderly patients.

Cohort and models		AUROC^b^ (95% CI)	Cutoff	Sensitivity (95% CI)	Specificity (95% CI)	PPV^c^ (95% CI)	NPV^d^ (95% CI)
**Training cohort**							
	LRM^e^	0.679 (0.660-0.698)	0.749	0.701 (0.670-0.732)	0.575 (0.556-0.594)	0.353 (0.331-0.376)	0.853 (0.836-0.869)
	XGBoost^f^	0.756 (0.740-0.773)	0.751	0.787 (0.758-0.813)	0.605 (0.586-0.624)	0.397 (0.374-0.421)	0.895 (0.880-0.909)
	LightGBM^g^	0.844 (0.831-0.857)	0.724	0.788 (0.759-0.814)	0.761 (0.745-0.777)	0.522 (0.495-0.549)	0.915 (0.903-0.927)
	MLP^h^	0.734 (0.717-0.751)	0.774	0.806 (0.778-0.832)	0.539 (0.520-0.558)	0.367 (0.345-0.389)	0.894 (0.877-0.908)
	RF^i^	0.814 (0.800-0.828)	0.748	0.810 (0.783-0.836)	0.671 (0.653-0.689)	0.449 (0.425-0.474)	0.914 (0.901-0.927)
	KNN^j^	0.712 (0.694-0.730)	0.789	0.742 (0.712-0.771)	0.558 (0.539-0.577)	0.357 (0.335-0.380)	0.867 (0.850-0.883)
**Internal validation cohort**							
	LRM	0.669 (0.650-0.688)	0.677	0.710 (0.679-0.740)	0.566 (0.547-0.585)	0.356 (0.333-0.378)	0.853 (0.835-0.869)
	XGBoost	0.684 (0.665-0.703)	0.657	0.614 (0.582-0.647)	0.663 (0.645-0.681)	0.381 (0.356-0.407)	0.836 (0.820-0.852)
	LightGBM	0.853 (0.841-0.865)	0.722	0.817 (0.791-0.842)	0.759 (0.742-0.775)	0.534 (0.507-0.560)	0.925 (0.913-0.936)
	MLP	0.719 (0.701-0.737)	0.739	0.751 (0.722-0.779)	0.587 (0.568-0.606)	0.380 (0.357-0.403)	0.875 (0.859-0.890)
	RF	0.823 (0.809-0.837)	0.745	0.844 (0.819-0.868)	0.653 (0.634-0.671)	0.450 (0.426-0.475)	0.926 (0.913-0.937)
	KNN	0.692 (0.674-0.711)	0.789	0.731 (0.701-0.760)	0.552 (0.532-0.571)	0.355 (0.333-0.377)	0.859 (0.841-0.875)
**External validation cohort**							
	LRM	0.763 (0.707-0.818)	0.787	0.830 (0.738-0.899)	0.586 (0.512-0.658)	0.503 (0.422-0.584)	0.872 (0.800-0.925)
	XGBoost	0.736 (0.678-0.794)	0.825	0.809 (0.714-0.882)	0.613 (0.539-0.683)	0.514 (0.430-0.596)	0.864 (0.793-0.917)
	LightGBM	0.755 (0.699-0.811)	0.899	0.851 (0.763-0.916)	0.597 (0.523-0.668)	0.516 (0.435-0.597)	0.888 (0.819-0.937)
	MLP	0.724 (0.665-0.784)	0.764	0.702 (0.599-0.792)	0.683 (0.611-0.749)	0.528 (0.437-0.618)	0.819 (0.750-0.876)
	RF	0.749 (0.692-0.806)	0.778	0.798 (0.702-0.874)	0.645 (0.572-0.714)	0.532 (0.446-0.616)	0.863 (0.795-0.916)
	KNN	0.632 (0.566-0.699)	0.789	0.670 (0.566-0.764)	0.527 (0.453-0.600)	0.417 (0.338-0.500)	0.760 (0.677-0.831)

^a^AKD: acute kidney disease.

^b^AUROC: area under the receiver operating characteristic curve.

^c^PPV: positive predictive value.

^d^NPV: negative predictive value.

^e^LRM: logistic regression model.

^f^XGBoost: Extreme Gradient Boosting.

^g^LightGBM: Light Gradient Boosting Machine.

^h^MLP: multilayer perceptron.

^i^RF: random forest.

^j^KNN: K-nearest neighbor.

#### Model Interpretability

**Figure 3:**
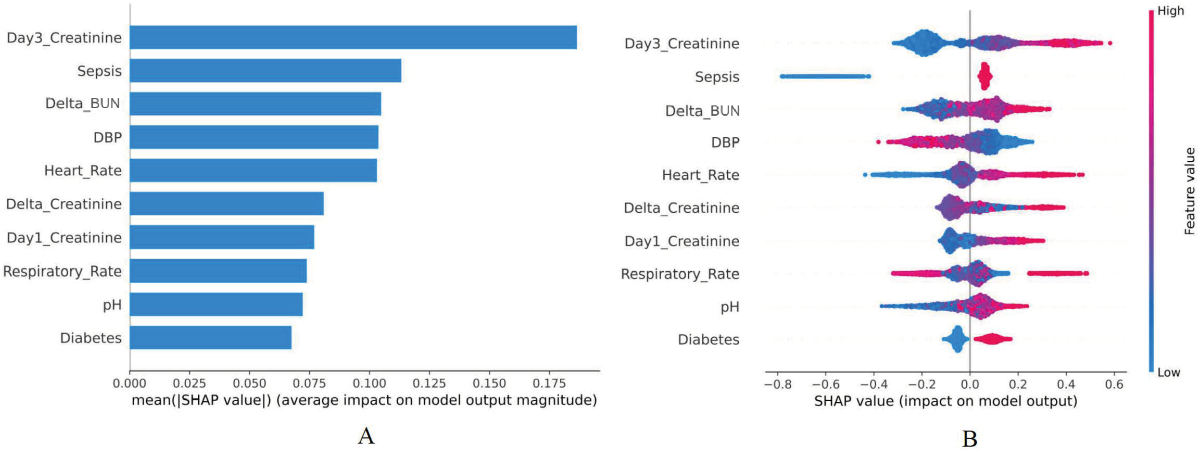
Feature-ranking plots (A) and summary plots (B) of LightGBM for predicting AKD risk. AKD: acute kidney disease; BUN: blood urea nitrogen; DBP: diastolic blood pressure; LightGBM: Light Gradient Boosting Machine; SHAP: Shapley additive explanation.

**Figure 4:**
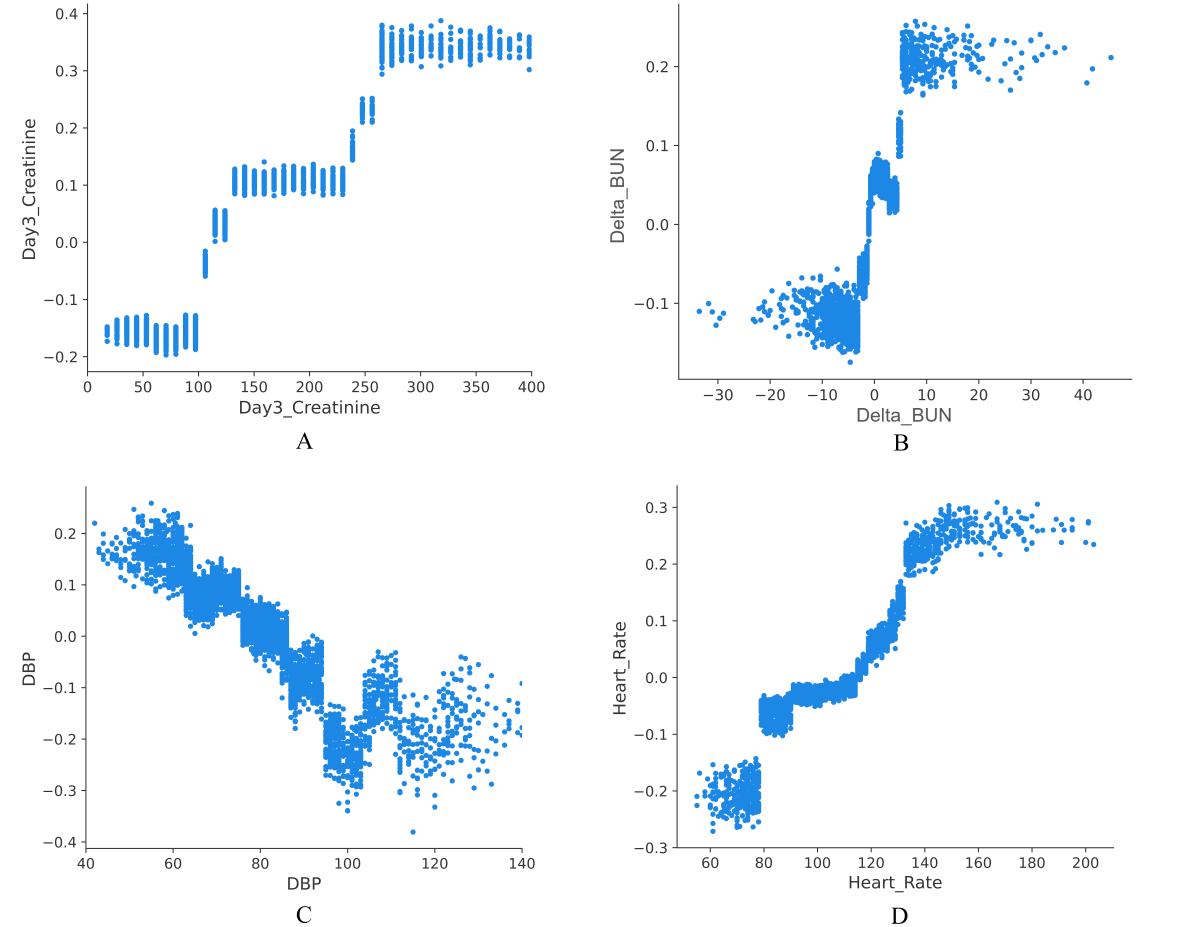
Partial dependence plots of LightGBM model for predicting AKD risk. AKD: acute kidney disease; BUN: blood urea nitrogen; DBP: diastolic blood pressure; LightGBM: Light Gradient Boosting Machine.

To better explain the clinical significance of certain features, this study quantified the features’ importance as SHAP values. As shown in Figure 3A, variables were given a ranking based on their contribution to the risk prediction of AKD, with creatinine on day 3, sepsis, delta BUN, DBP, heart rate, delta creatinine, creatinine on day 1, respiratory rate, pH, and diabetes as the top 10 predictors of developing AKD during hospitalization in the elderly. Figure 3B shows a detailed relationship between each feature and AKD risk, indicating that the positively related features were as follows: creatinine on day 3, sepsis, delta BUN, heart rate, delta creatinine, creatinine on day 1, pH, and diabetes (the higher the value of these features or the presence of complications, the higher the probability of developing AKD in elderly patients with AKI). Further, the protective effect was associated with a higher DBP. However, the relationship between respiratory rate and AKD during hospitalization was not clearly demonstrated. Furthermore, partial dependence plots were drawn in Figure 4 for the first 4 continuous variables in Figure 3A. The partial dependence plots visually displayed the global relationship between feature and risk distribution. According to Figure 4A, the change curve between creatinine on day 3 (abscissa) and AKD risk (ordinate) indicated a cutoff value of 110 for this feature, meaning that when the creatinine level on day 3 exceeded 110 umol/L, the risk of AKD during hospitalization also increased. Similarly, Figures 4B, 4C, and 4D demonstrate that 0 was the cutoff for delta BUN (positive correlation), 80 mmHg for the DBP (negative correlation), and 110 beats/minute for the heart rate (positive correlation). Thus, targeted feature management may assist in reducing the risk of AKD in elderly patients with AKI during hospitalization based on the cutoff values in partial dependency plots.

#### Model Application

We deployed the LightGBM algorithm as an online app because the LightGBM AKD risk model had a relatively high AUROC in the training, internal validation, and external cohorts. After the 10-fold cross-validation grid search, the LightGBM hyperparameters were finally tuned as follows: “num_leaves”: 10, “max_depth”: 5, “max_bin”: 135, “min_data_in_leaf”: 11, “feature_fraction”: 1.0, “bagging_fraction”: 1.0, “bagging_freq”: 45, “lambda_l1”: 0.0, “lambda_l2”: 0.001, “min_split_gain”: 0.4. Further, a web-based app for predicting AKD risk in the elderly was designed, which could be accessed online at any time by medical staff or patients (Multimedia Appendix 5) [19]. For an elderly patient with AKI being diagnosed for the first time in the ICU, physicians collected and input all variables’ values correctly in Multimedia Appendix 5 and then clicked the Predict button to obtain the predicted result (AKD or non-AKD) during hospitalization. Moreover, users could enter variables’ values and the author’s email address and click the Feedback button, enabling new data to be sent to the author to facilitate model iteration. When the result showed that the patient was at high risk of AKD, early intervention could be implemented based on the partial dependence plots in Figure 4 and interventionable indicators might be controlled as close to the cutoff value as possible to prevent the progression of AKI and reduce the risk of AKD.

### Model 2 : Predicting Prognostic Mortality in Elderly Patients With AKD

#### Baseline Characteristics

In this study on predicting the prognostic mortality in elderly patients with AKD, a total of 2661 elderly patients with AKD from the MIMIC-IV database (training cohort) and 186 from Xiangya Hospital (external validation cohort) were screened out and enrolled. The in-hospital mortality of elderly patients with AKD was 29.6% (788/2661) in the training cohort and 41.3% (77/186) in the external validation cohort. Multimedia Appendix 6 provides the differences in baseline characteristics between the 2 cohorts stratified by in-hospital death. In the MIMIC-IV cohort, compared with survivors, patients who died in the hospital were more likely to be older (*P*<.05), with a higher proportion of comorbidities (sepsis, CKD, and CLDl *P*<.05); a higher proportion of interventions (RRT and vasopressor use; *P*<.05); a higher heart rate and lower SBP in terms of vital signs (*P*<.05); and higher WBC count, potassium, anion gap, glucose, BUN on day 1, creatinine on day 1, BUN on day 3, creatinine on day 3, delta BUN, and delta creatinine and lower RBC count, hemoglobin, hematocrit, and PaCO_2_ in terms of laboratory tests (*P*<.05). Additionally, in the Xiangya Hospital cohort, sepsis, RRT, vasopressor use, heart rate, anion gap, and BUN on day 1 had similar statistical trends to those in the MIMIC-IV cohort (*P*<.05).

#### Model Comparison

**Figure 5:**
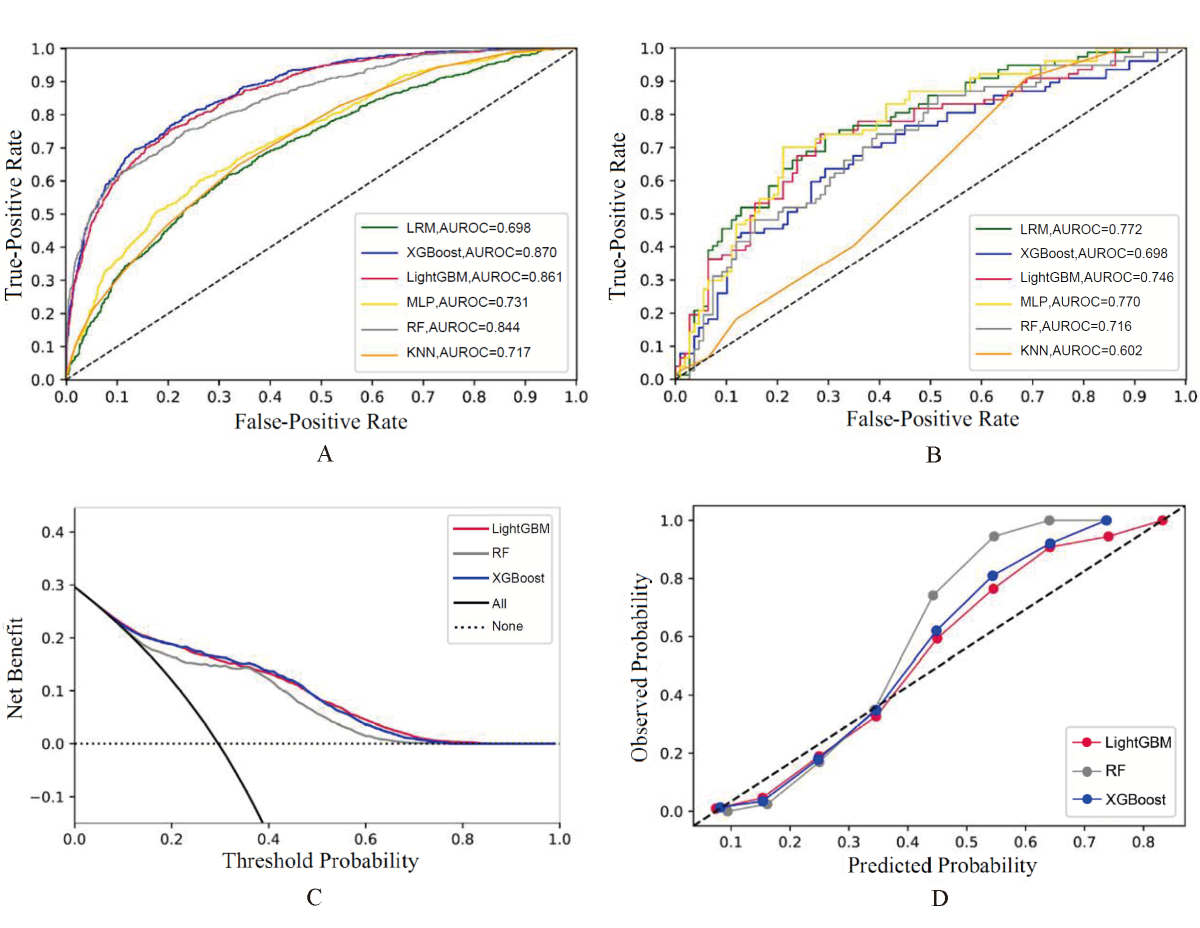
ROC curves, DCA , and calibration curves of AKD prognostic mortality prediction models. AKD: acute kidney disease; AUROC: area under the receiver operating characteristic curve; DCA: decision curve analysis; KNN: K-nearest neighbor; LightGBM: Light Gradient Boosting Machine; LRM: logistic regression model; MLP: multilayer perceptron; RF: random forest; ROC: receiver operating characteristic; XGBoost: Extreme Gradient Boosting.

The performance of the in-hospital death prediction model for AKD in elderly patients in the training and external cohorts is presented in Table 2. In the training cohort, the best-performing algorithm was XGBoost, with AUROC=0.870 (95% CI 0.853-0.886), sensitivity=0.772 (95% CI 0.752-0.791), specificity=0.793 (95% CI 0.763-0.821), PPV=0.594 (95% CI 0.564-0.624), and NPV=0.899 (95% CI 0.883-0.913). In the external validation cohort, the LRM provided the best prediction, with AUROC=0.772 (95% CI 0.701-0.843), sensitivity=0.706 (95% CI 0.612-0.790), specificity=0.740 (95% CI 0.628-0.834), PPV=0.640 (95% CI 0.532-0.739), and NPV=0.794 (95% CI 0.700-0.869). However, comprehensively comparing the prediction performance of the training cohort and the generalization of the validation cohort, the LightGBM algorithm showed good overall performance, as demonstrated by an AUROC of 0.861 (95% CI 0.843-0.878) in the training cohort, 0.868 (95% CI 0.851-0.885) in the internal validation cohort, and 0.746 (95% CI 0.673-0.820) in the external cohort, in accordance with the ROC curves in Figure 5A, Multimedia Appendix 7, and Figure 5B. Figure 5C indicates that in DCA, when the probability of death during hospitalization reached 10%, the net benefit of intervention measures was 0.2, suggesting good efficacy of LightGBM. Moreover, the calibration curve in Figure 5D shows that the predicted curve of the model surrounded the actual probability line, indicating relative accuracy.

**Table 2:** Performance of the AKD^a^ prognostic mortality models for elderly patients.

Cohort and models			AUROC^b^ (95% CI)		Cutoff		Sensitivity (95% CI)		Specificity (95% CI)		PPV^c^ (95% CI)		NPV^d^ (95% CI)
**Training cohort**													
	LRM^e^	0.698 (0.675-0.721)		0.308		0.683 (0.661-0.704)		0.619 (0.584-0.653)		0.451 (0.421-0.481)		0.810 (0.790-0.829)	
	XGBoost^f^	0.870 (0.853-0.886)		0.312		0.772 (0.752-0.791)		0.793 (0.763-0.821)		0.594 (0.564-0.624)		0.899 (0.883-0.913)	
	LightGBM^g^	0.861 (0.843-0.878)		0.334		0.798 (0.779-0.816)		0.754 (0.722-0.784)		0.610 (0.579-0.641)		0.885 (0.869-0.899)	
	MLP^h^	0.731 (0.709-0.753)		0.332		0.737 (0.716-0.757)		0.603 (0.568-0.637)		0.491 (0.459-0.523)		0.815 (0.796-0.833)	
	RF^i^	0.844 (0.826-0.862)		0.351		0.887 (0.872-0.901)		0.632 (0.597-0.666)		0.702 (0.667-0.736)		0.851 (0.835-0.867)	
	KNN^j^	0.717 (0.695-0.740)		0.313		0.662 (0.640-0.683)		0.648 (0.614-0.682)		0.447 (0.418-0.476)		0.817 (0.797-0.837)	
**Internal validation cohort**													
	LRM	0.720 (0.697-0.742)		0.301		0.676 (0.642-0.709)		0.669 (0.647-0.690)		0.449 (0.420-0.479)		0.838 (0.818-0.856)	
	XGBoost	0.810 (0.790-0.830)		0.290		0.793 (0.763-0.822)		0.686 (0.665-0.707)		0.503 (0.474-0.532)		0.893 (0.876-0.908)	
	LightGBM	0.868 (0.851-0.885)		0.303		0.828 (0.799-0.854)		0.753 (0.733-0.773)		0.573 (0.543-0.602)		0.916 (0.901-0.929)	
	MLP	0.750 (0.728-0.771)		0.339		0.633 (0.598-0.667)		0.744 (0.724-0.764)		0.497 (0.465-0.529)		0.835 (0.817-0.853)	
	RF	0.706 (0.605-0.759)		0.330		0.625 (0.561-0.637)		0.761 (0.745-0.776)		0.567 (0.533-0.599)		0.776 (0.760-0.790)	
	KNN	0.725 (0.703-0.748)		0.278		0.762 (0.730-0.792)		0.558 (0.535-0.580)		0.408 (0.382-0.434)		0.854 (0.833-0.873)	
**External validation cohort**													
	LRM	0.772 (0.701-0.843)		0.285		0.706 (0.612-0.790)		0.740 (0.628-0.834)		0.640 (0.532-0.739)		0.794 (0.700-0.869)	
	XGBoost	0.698 (0.620-0.776)		0.385		0.706 (0.612-0.790)		0.636 (0.519-0.743)		0.605 (0.490-0.712)		0.733 (0.638-0.815)	
	LightGBM	0.746 (0.673-0.820)		0.312		0.716 (0.621-0.798)		0.740 (0.628-0.834)		0.648 (0.539-0.747)		0.796 (0.703-0.871)	
	MLP	0.770 (0.699-0.841)		0.339		0.789 (0.700-0.861)		0.701 (0.586-0.800)		0.701 (0.586-0.800)		0.789 (0.700-0.861)	
	RF	0.716 (0.639-0.792)		0.337		0.606 (0.507-0.698)		0.740 (0.628-0.834)		0.570 (0.467-0.669)		0.767 (0.664-0.852)	
	KNN	0.602 (0.519-0.685)		0.188		0.312 (0.227-0.408)		0.909 (0.822-0.963)		0.483 (0.399-0.567)		0.829 (0.679-0.928)	

^a^AKD: acute kidney disease.

^b^AUROC: area under the receiver operating characteristic curve.

^c^PPV: positive predictive value.

^d^NPV: negative predictive value.

^e^LRM: logistic regression model.

^f^XGBoost: Extreme Gradient Boosting.

^g^LightGBM: Light Gradient Boosting Machine.

^h^MLP: multilayer perceptron.

^i^RF: random forest.

^j^KNN: K-nearest neighbor.

#### Model Interpretability

**Figure 6:**
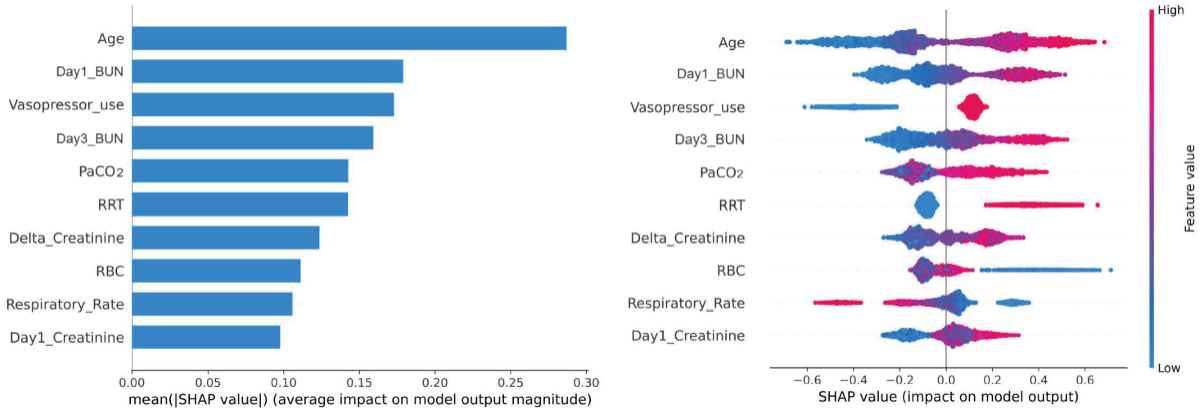
Feature-ranking plots (A) and summary plots (B) of LightGBM for predicting prognostic mortality in AKD. AKD: acute kidney disease; BUN: blood urea nitrogen; PaCO_2_: partial pressure of carbon dioxide; LightGBM: Light Gradient Boosting Machine; RBC: red blood cell; RRT: renal replacement therapy; SHAP: Shapley additive explanation.

**Figure 7:**
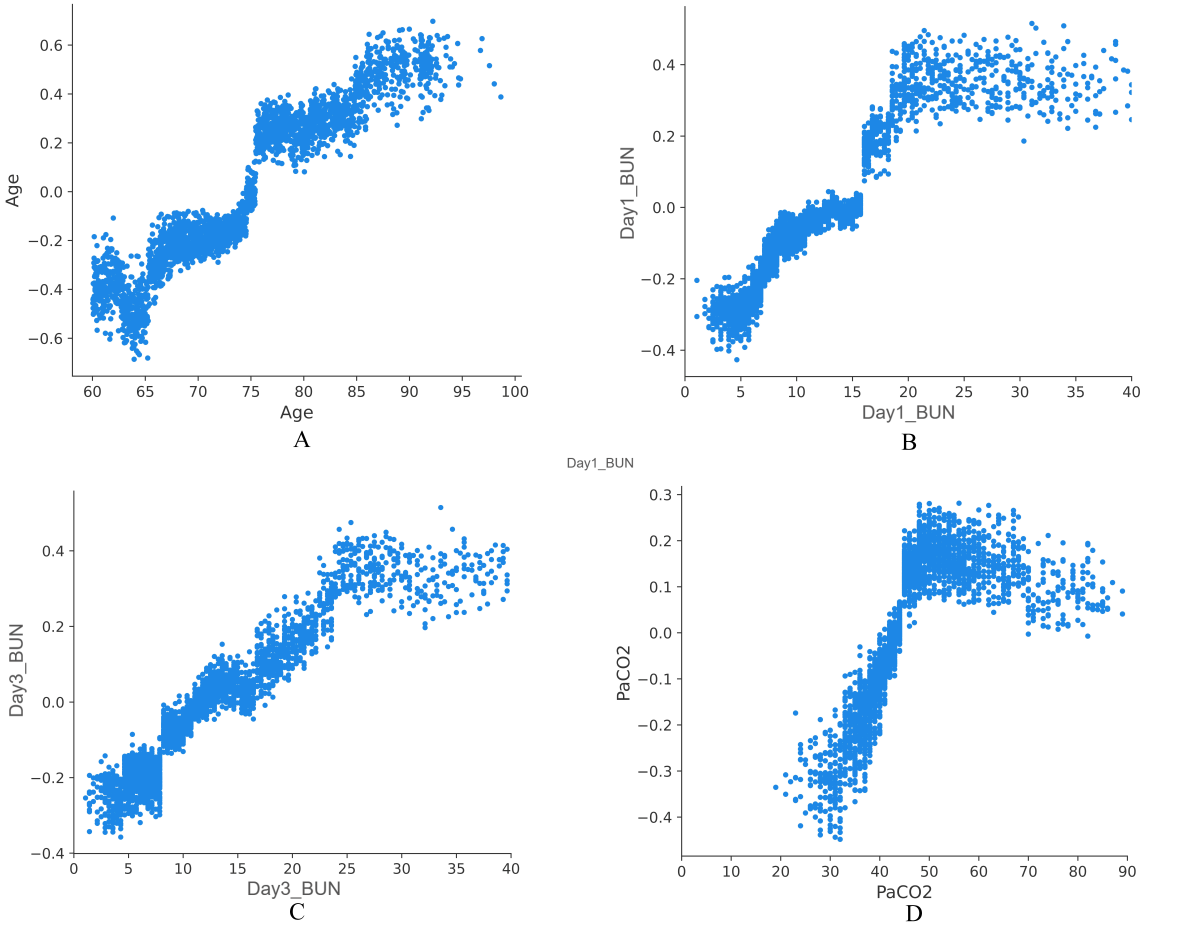
Partial dependence plots of LightGBM for predicting prognostic mortality in patients with AKD. AKD: acute kidney disease; BUN: blood urea nitrogen; LightGBM: Light Gradient Boosting Machine; PaCO_2_: partial pressure of carbon dioxide.

Using SHAP values, we performed a visual analysis of a model for predicting AKD prognostic mortality of the elderly. Figure 6A shows the top 10 predictors of in-hospital death in patients with AKD, as follows: age, BUN on day 1, vasopressor use, BUN on day 3, PaCO_2_, RRT, delta creatinine, RBC count, respiratory rate, and creatinine on day 1. Figure 6B indicates a more detailed representation of the positive and negative relationships between features and outcomes. The risk of death due to AKD during hospitalization was positively associated with the following features: older age, higher BUN on day 1, use of vasopressors, higher BUN on day 3, higher PaCO_2_, use of RRT, higher delta creatinine, and creatinine on day 1. The RBC count and respiratory rate were higher in hospitalized survivors among elderly patients with AKD. For the first 4 continuous variables in Figure 6A, partial dependence plots were drawn (Figure 7). According to Figure 7A, the probability of in-hospital death increased from 0 when the patient reached 75 years of age. Similarly, Figures 7B, 7C, and 7D show that the cutoff values of BUN on day 1, BUN on day 3 and PaCO_2_ affecting the risk of death were 15 mmol/L, 10 mmol/L, and 45 mmHg, respectively, which might contribute to guiding patients’ management and reducing the in-hospital risk of death for those with AKD.

#### Model Application

We chose the LightGBM algorithm, which exhibited good AUROC values in the training and validation cohorts, to deploy the prognostic mortality function in the online version of the AKD model. The optimal combination of hyperparameters for the LightGBM prognostic model was as follows: “num_leaves”: 10, “max_depth”: 4, “max_bin”: 35, “min_data_in_leaf”: 100, “feature_fraction”: 1.0, “bagging_fraction”: 0.7, “bagging_freq”: 5, “lambda_l1”: 0.0, “lambda_l2”: 0.1, “min_split_gain”: 0.0. The web-based app to predict in-hospital death in elderly patients with AKD could be accessed online (Multimedia Appendix 8) [20]. When elderly patients were diagnosed with AKD for the first time, we entered all the indicators correctly on the web page and clicked the Predict button to predict the prognosis (death or survival) of elderly patients with AKD during hospitalization. Additionally, if users found a prediction error, they could enter the variable’s value and their own email address and then click the Feedback button, enabling the corresponding data to be automatically sent to the author’s email address. Using this feedback function could facilitate the collection of new data for model iterations. At the same time, according to the cutoff value shown in the partial dependence plots in Figure 7, targeted interventions were performed on patients at risk of death due to AKD, with the potential to improve the survival of patients with AKD.

## Discussion

### Principal Findings

#### Predicting the Risk of AKD in the Elderly

As part of this study, we focused on model construction and feature analysis for AKD risk during hospitalization, and LightGBM was selected as the best algorithm for online deployment (training cohort AUROC=0.844, 95% CI 0.831-0.857; validation cohort AUROC=0.755, 95% CI 0.699-0.811). To the best of our knowledge, our study was the first to analyze the risk characteristics of AKD in critically ill elderly patients during hospitalization and to develop an easy-to-use online AKD risk identification app.

In addition to basic information, comorbidities, vital signs, and laboratory indicators on day 1 of AKI diagnosis, some indicators on day 3 and their fluctuations were also selected as features, including creatinine on day 3, BUN on day 3, delta BUN, and delta creatinine. A previous study found that the maximum creatinine level is reached on day 3 within 1-5 days after cardiac surgery in elderly patients [21]. Treiber et al [22] demonstrated that in neonatal patients with perinatal hypoxia, the AUROC of serum creatinine on day 3 after birth as a single predictor for AKI is 0.660, indicating a certain predictive value. Similar to these studies, our study revealed that serum creatinine on day 3 is higher than that on day 1 of AKD diagnosis in the elderly, ranking first in the feature of the AKD risk prediction model. Thus, serum creatinine on day 3 might be considered a focused experimental indicator for clinical research on patients with AKD in the ICU.

Delta BUN is commonly used to evaluate changes in renal function; however, the definition of the specific delta BUN varies. According to a study on patients with acute heart failure, delta BUN refers to the difference between the day before and after the administration of loop diuretics, but there was no statistical difference between the treatment and control groups (*P*>.05) [23]. Moreover, delta BUN was defined as the difference between 1 year after transplantation and at transplantation to evaluate renal function in a retrospective study conducted by Ewald et al [24]. In our study, we found that delta BUN (day 3 – day 1 after AKI diagnosis) is significantly positively correlated with AKD in elderly patients, with higher BUN on day 3 than on day 1 (delta BUN>0). Wu et al [25] also observed a gradual increase in BUN after AKI, in which BUN peaked at day 3 following cisplatin-induced AKI. Additionally, we determined that delta creatinine and creatinine on day 1 are associated with an increased risk of AKD during hospitalization. In a prospective study on adult patients after cardiac surgery, researchers defined delta creatinine as baseline – first postoperative creatinine and concluded that delta creatinine combined with biomarkers has a good predictive effect on mortality[24]. Furthermore, Garner et al [26] defined delta creatinine to be higher than 26 μmol/L within 30 days of admission, enabling 98% of hospitalized patients with AKI to be identified.

At present, many studies have examined the factors associated with sepsis-related AKI, such as age, CKD, diabetes, infective endocarditis, and intra-abdominal infections [27-30]. However, there are relatively few studies conducted on sepsis and AKD. According to a single-center retrospective study, 46.9% of patients with sepsis developed AKD; in other words, sepsis is a critical factor contributing to the development of AKD in patients with AKI [31]. As a result of this study, it was also found that sepsis has a significant influence on renal function recovery of elderly patients with AKI.

Renal dysfunction is primarily caused by insufficient renal perfusion, indicating that improving the patient’s hemodynamics to increase perfusion pressure might be an effective strategy for reversing kidney damage [32]. Previously, it was demonstrated that the DBP might be a valuable target for hemodynamic therapy in AKI by affecting renal perfusion [33]. Additionally, we found that the DBP is a major factor in the occurrence of AKD in elderly patients with AKI and that the risk of AKD gradually increases as the DBP decreases from 80 mmHg. However, a study of patients with severe coronary artery disease found that the risk of AKD is higher when the DBP is less than 50 mmHg [34]. This difference in the cutoff of the DBP for predicting AKD might reflect varying patient populations. As an indicator of overall health, the heart rate is affected by many factors, including pain stimulation, temperature, blood volume, and inflammatory responses. In a randomized controlled trial of β-receptor blockers in heart failure, maintaining a heart rate of 60 beats/minute was found to be beneficial to patient outcomes [35]. Additionally, a heart rate higher than 100 beats/minute might be a predictor of sepsis in patients not on advanced life support [36]. Our study also revealed that AKD is more likely to develop when the heart rate exceeds 110 beats/minute.

There is evidence that an abnormal respiratory rate could interfere with the baroreceptor reflex and cardiovascular variability [37]. We also found that an excessively high or low respiratory rate might adversely affect renal function recovery and lead to AKD in elderly patients with AKI. Metabolic acidosis is a common and life-threatening homeostatic disorder in the ICU, especially in patients with sepsis [38]. Furthermore, acidosis-related hemodynamic changes and decreased pH also contribute to the risk of AKI [39]. However, there has been relatively little attention given to metabolic alkalosis resulting from mass gastric fluid loss, a compensatory response to respiratory acidosis, or excess diuresis in critically ill patients. In a retrospective study of patients with septic shock, metabolic alkalosis was a significant predictor of the length of stay [40]. Likewise, we found that elevated pH is also a predictor of patients with AKD in the ICU, suggesting persistent renal impairment. Diabetes as a chronic disease is preventable and controllable. Currently, some studies have indicated that AKI is more common among patients with diabetes and that diabetes might increase AKI risk [41]. According to a national study of hospitalization trends in AKI in the United States between 2000 and 2014, the incidence of AKI among patients with diabetes was significantly higher than among patients without diabetes [42]. Our study also demonstrated that diabetes contributes to the development of AKD in elderly patients with AKI in the ICU.

#### Predicting Prognostic Mortality in Elderly Patients With AKD

After analyzing and predicting the risk of AKD during hospitalization for elderly patients with AKI in the ICU, a further machine learning prediction study was conducted on the hospital prognostic mortality of patients with AKD. Finally, the LightGBM algorithm was selected and deployed as a user-friendly web app, which performed well in both the training (AUROC=0.861, 95% CI 0.843-0.878) and external validation (AUROC=0.746, 95% CI 0.673-0.820) cohorts. In our opinion, this study was the first to construct and validate online machine learning models for continuously predicting the AKD risk and prognostic mortality in elderly patients.

Notably, we found that among the top 10 significant variables for predicting the occurrence of AKD in patients with AKI and predicting the prognostic mortality in AKD, delta creatinine, creatinine on day 1, and respiratory frequency all had good predictive values. In the prediction of hospital death in the elderly with AKD, creatinine on day 1 following renal injury was proportional to the likelihood of death. Some studies have shown that serum creatinine and mortality risk are significantly correlated. Thongprayoon et al [43] concluded that the serum creatinine level is a reliable predictor of mortality in critically ill patients. According to a retrospective study by Pooja et al [44], hepatorenal syndrome–related death is independently affected by high serum creatinine levels. Further, our study observed a positive correlation between delta creatinine and hospital mortality in elderly patients with AKD, which also appeared relevant to the risk of developing AKD in elderly patients with AKI. In patients with liver cirrhosis, researchers have found that creatinine variability (ie, delta creatinine) can serve as an effective indicator for predicting mortality [45]. Bradypnea is often seen in patients with central respiratory failure, sleep apnea syndrome, and high intracranial pressure. There is evidence to suggest that slow breathing can lead to death in patients with many diseases, including traumatic brain injury and stroke [46,47]. Additionally, Gooneratne et al [48] revealed that slow breathing with drowsiness results in increased mortality in the elderly. In this study, we also found that slow breathing is highly predictive of mortality in elderly patients with AKD.

In previous studies, age has been found to be a critical factor in the development of AKI [49]. Further, a retrospective study of Chinese multicenter patients with AKI revealed that age is an independent predictor of AKD progression in the logistic regression model but is not considered a risk factor for death [9]. As found in our study, age ranked first among the factors influencing in-hospital death in elderly patients with AKD, with patients aged over 75 years showing higher mortality and those 60-75 years old not appearing to present a significant risk of death. Patients in the ICU with septic shock or cardiogenic shock usually require vasopressors, such as norepinephrine, epinephrine, and dopamine. Plurad et al [50] found that early administration of vasopressors in the ICU is independently associated with the risk of death regardless of the volume status at admission. In our study, vasopressor use was also a key predictor of mortality in elderly patients with AKD. Research has indicating that BUN is closely related to mortality risk in those with critical illness, acute pancreatitis, and heart failure [51-53]. In our study, we found that BUN on day 1 and day 3 after AKI diagnosis contributes to the risk of death due to AKD. Importantly, the cutoff values for BUN on day 1 and day 3 to predict in-hospital death were determined to be 15 and 20 mmol/L, respectively. However, Wernly et al [54] calculated 9.7 mmol/L as the optimal cutoff for BUN using Youden’s index to predict patients’ mortality in the ICU. Among patients with acute myocardial infarction complicated by cardiogenic shock, Zhu et al [55] observed that patients with BUN levels higher than 8.95 mmol/L on admission have an adverse short-term outcome [55].

As the main indicator of respiratory health and acid-base homeostasis, PaCO_2_ levels higher than 45 mmHg often indicate the presence of hypercapnia. In a prospective observational study, patients with hypercapnia experienced higher in-hospital and long-term mortality [56]. Additionally, we observed that elderly patients with AKD who had PaCO_2_ levels higher than 45 mmHg are more likely to die during hospitalization. RRT has been widely used as an effective intervention in patients with AKI, acute severe pancreatitis, and poisoning. Our study suggested that elderly patients with AKD requiring RRT might be at higher risk of in-hospital death, similar to the fact that patients with AKI in need for RRT usually have poorer survival and less renal function recovery, although RRT could delay or even stop this adverse process [57]. Based on our findings, AKD is associated with decreased RBC counts, which are observed in aplastic anemia, iron deficiency anemia, and massive bleeding. Recently, the RBC distribution width (RDW) has been widely regarded as a predictor of prognosis, especially in patients with coronary heart disease, AKI, and CKD [58-60]. Nevertheless, because of the high missing proportion of the RDW, we did not include it in our constructed model.

### Strengths

We focused on elderly individuals with AKD for the first time, identified features affecting AKD risk and prognostic mortality, and developed 2 web-based prediction apps. After the users input the apps’ URLs on the mobile phone or computer and manually entered the variables’ values, they could click the Predict button to obtain the predictions or the Feedback button to send us new data. Although our online apps are easy to use, the calibration tool deployed by Sun et al [61] is more convenient and can be automated for use at different hospitals without manual data preparation, which could serve as a reference for further iterative development. Of note, data sets from Xiangya Hospital (China) were used for external validation, with good performance. However, the performance of the AKI prediction model in different sites has shown significant degradation [61], which might be due to the following measures we took to minimize performance degradation caused by a data shift. First, we adopted relatively strict inclusion and exclusion criteria to reduce the heterogeneity of enrolled patients. In addition, AKI and AKD were defined based on laboratory measurements, which prevented errors in medical record text recognition. Third, the units of the variables in the MIMIC-IV and Xiangya Hospital cohorts were unified. Finally, we added the Feedback button to 2 online forecasting apps to gather new training data through user feedback, that is, coping with data shifts by adhering to the fundamental principle of increasing training data.

### Limitations

However, there are still some limitations. First, since detailed information about patients after discharge was lacking, the emphasis was placed on AKD diagnosis and prognosis during hospitalization. Second, the prediction models were based on machine learning classification algorithms, which could only identify a high or a low risk of AKD and patients’ survival but could not display detailed risk values. Finally, although the 2 prediction models constructed in this study were externally validated and demonstrated good generalization abilities, additional variables, such as biomarkers, were needed to ensure better performance, as well as prospective experiments to further evaluate the online apps.

### Conclusion

In conclusion, 2 online apps with machine learning algorithms were successfully constructed and deployed for predicting the AKD risk and prognostic mortality in elderly patients. SHAP can intuitively explain the rankings of importance, threshold values for partial features, and positive or negative correlations between features and outcomes, thereby aiding medical staff in early identification and targeted management to promote renal function recovery and patient survival to a certain extent.

## Appendix

Multimedia Appendix 1 The timeline plot of AKI and AKD during hospitalization. AKD: acute kidney disease; AKI: acute kidney injury.

Multimedia Appendix 2 The optimal hyperparameters.

Multimedia Appendix 3 The characteristics of elderly patients with AKI. AKI: acute kidney injury.

Multimedia Appendix 4 The ROC curves in the internal validation of AKD risk prediction models.

Multimedia Appendix 5 The internet-based app of the LightGBM model for predicting the AKD risk. AKD: acute kidney disease; LightGBM: Light Gradient Boosting Machine.

Multimedia Appendix 6 The characteristics of elderly patients with AKD. AKD: acute kidney disease.

Multimedia Appendix 7 The ROC curves in the internal validation of prognostic mortality prediction models.

Multimedia Appendix 8 The internet-based app of the LightGBM model for predicting AKD mortality. AKD: acute kidney disease; LightGBM: Light Gradient Boosting Machine.

## Abbreviations

ADQI: Acute Disease Quality Initiative
AI: artificial intelligence
AKD: acute kidney disease
AKI: acute kidney injury
AUROC: area under the receiver operating characteristic curve
BUN: blood urea nitrogen
CKD: chronic kidney disease
CLD: chronic liver disease
CPD: chronic pulmonary disease
DBP: diastolic blood pressure
DCA: decision curve analysis
EHR: electronic health record
ESRD: end-stage renal disease
ICU: intensive care unit
KDIGO: Kidney Disease: Improving Global Outcomes
KNN: K-nearest neighbor
LightGBM: Light Gradient Boosting Machine
LRM: logistic regression model
MIMIC-IV: Medical Information Mart for Intensive Care IV
MLP: multilayer perceptron
MV: mechanical ventilation
NPV: negative predictive value
PPV: positive predictive value
PaCO_2_: partial pressure of carbon dioxide
PaO2: partial pressure of oxygen
RBC: red blood cell
RDW: RBC distribution width
RF: random forest
ROC: receiver operating characteristic
RRT: renal replacement therapy
SBP: systolic blood pressure
SHAP: Shapley additive explanation
WBC: white blood cell
XGBoost: Extreme Gradient Boosting

## Data Availability

The data sets used and analyzed during this study are available from the corresponding author upon reasonable request.
