# Supplementary material for: Machine Learning for Predicting Risk and Prognosis of Acute Kidney Disease in Critically Ill Elderly Patients During Hospitalization: Internet-Based and Interpretable Model Study
Source: J Med Internet Res. 2024 May 1;26:e51354. doi: 10.2196/51354 (PMC11097053; doi:10.2196/51354)
Supplement: Multimedia Appendix 1 [file jmir_v26i1e51354_app1.pdf]

Multimedia Appendix 1. The timeline plot of AKI and AKD during hospitalization.

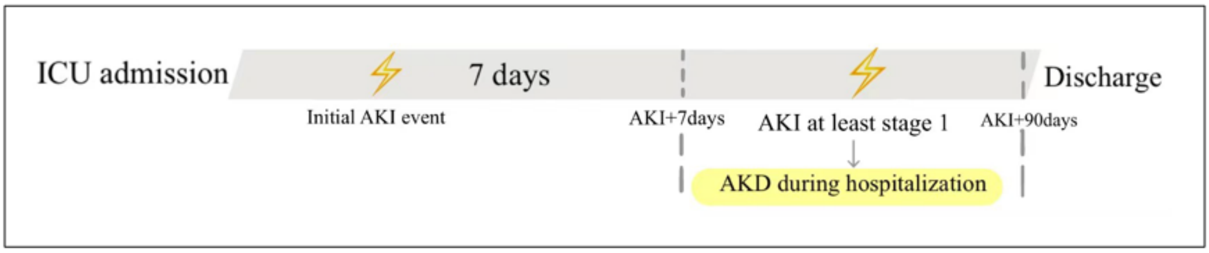

AKI, acute kidney injury; AKD, acute kidney disease.

AKD was defined as the presence of AKI at least stage 1 within 7 to 90 days after the initial AKI event. We defined the patients with AKD who met this definition before discharge from hospital as AKD during hospitalization.
