# Supplementary material for: Machine Learning for Predicting Risk and Prognosis of Acute Kidney Disease in Critically Ill Elderly Patients During Hospitalization: Internet-Based and Interpretable Model Study
Source: J Med Internet Res. 2024 May 1;26:e51354. doi: 10.2196/51354 (PMC11097053; doi:10.2196/51354)
Supplement: Multimedia Appendix 2 [file jmir_v26i1e51354_app2.pdf]

Multimedia Appendix 2.

A. The optimal hyperparameters for AKD risk models based on the ten-fold cross-validation.

Algorithms	Hyperparameters	Settings
**LRM**	C<br>max_iter<br>solver	0.00285<br>100000<br>newton-cg
**XGBoost**	gamma<br>max_depth<br>min_child_weight<br>max_delta_step	0.1<br>4<br>None<br>None
**LightGBM**	num_leaves<br>max_depth<br>max_bin<br>min_data_in_leaf<br>feature_fraction<br>bagging_fraction<br>bagging_freq<br>lambda_l1<br>lambda_l2<br>min_split_gain	10<br>5<br>135<br>11<br>1.0<br>1.0<br>45<br>0<br>0.001<br>0.4
**MLP**	kernel_initializer<br>activation	uniform<br>sigmoid
**RF**	max_depth<br>max_features<br>min_samples_leaf<br>min_samples_split	6<br>10<br>4<br>12
**KNN**	metric<br>n_neighbors	manhattan<br>19

LRM, Logistic Regression Model; XGBoost, eXtremely Gradient Boosting; LightGBM, Light Gradient Boosting Machine; MLP, Multi-Layer Perceptron; RF, Random Forest; KNN, KNearest Neighbor.

B. The optimal hyperparameters for AKD prognostic mortality models based on the ten-fold cross-validation.

Algorithms	Hyperparameters	Settings
**LRM**	C<br>max_iter<br>solver	0.17074<br>100000<br>newton-cg
**XGBoost**	gamma<br>max_depth<br>min_child_weight<br>max_delta_step	0.2<br>3<br>None<br>None
**LightGBM**	num_leaves<br>max_depth<br>max_bin<br>min_data_in_leaf<br>feature_fraction<br>bagging_fraction<br>bagging_freq<br>lambda_l1<br>lambda_l2<br>min_split_gain	10<br>4<br>35<br>100<br>1.0<br>0.7<br>5<br>0.0<br>0.1<br>0.0
**MLP**	kernel_initializer<br>activation	uniform<br>sigmoid
**RF**	max_depth<br>max_features<br>min_samples_leaf<br>min_samples_split	8<br>11<br>4<br>12
**KNN**	metric<br>n_neighbors	minkowski<br>18

LRM, Logistic Regression Model; XGBoost, eXtremely Gradient Boosting; LightGBM, Light Gradient Boosting Machine; MLP, Multi-Layer Perceptron; RF, Random Forest; KNN, KNearest Neighbor.
