# Supplementary material for: Machine Learning for Predicting Risk and Prognosis of Acute Kidney Disease in Critically Ill Elderly Patients During Hospitalization: Internet-Based and Interpretable Model Study
Source: J Med Internet Res. 2024 May 1;26:e51354. doi: 10.2196/51354 (PMC11097053; doi:10.2196/51354)
Supplement: Multimedia Appendix 3 [file jmir_v26i1e51354_app3.pdf]

Multimedia Appendix 3. The characteristics of elderly patients with AKI.

Variables	MIMIC-IV cohort AKD (n=2661)	MIMIC-IV cohort Non-AKD (n=881)	*P*-value	Xiangya hospital cohort AKD (n=186)	Xiangya hospital cohort Non-AKD (n=94)	*P*-value
**Basic information**						
Age, years	73.5 [67.1, 80.9]	74.3 [67.4, 81.6]	0.187	72.0 [65.0, 79.0]	70.0 [66.0, 78.0]	0.460
Gender, n (%)			0.159			<0.001
Male	1515 (56.9)	477 (54.1)		143 (76.9)	51 (54.3)	
Female	1146 (43.1)	404 (45.9)		43 (23.1)	43 (45.7)	
Aki stage, n (%)			0.532			<0.001
I	2044 (76.8)	690 (78.3)		52 (28.0)	51 (54.3)	
II	568 (21.3)	173 (19.6)		45 (24.2)	27 (28.7)	
III	49 (1.8)	18 (2.0)		89 (47.8)	16 (17.0)	
**Comorbidities, n (%)**						
Sepsis	2457 (92.3)	749 (85.0)	<0.001	40 (21.5)	20 (21.3)	0.326
Hypertension	1334 (50.1)	491 (55.7)	0.004	134 (72.0)	49 (52.1)	0.002
Diabetes	1047 (39.3)	288 (32.7)	<0.001	72 (38.7)	15 (16.0)	<0.001
CKD	823 (30.9)	205 (23.3)	<0.001	86 (46.2)	9 (9.6)	<0.001
CPD	963 (36.2)	276 (31.3)	0.010	33 (17.7)	13 (13.8)	0.507
CLD	218 (8.2)	54 (6.1)	0.055	37 (19.9)	11 (11.7)	0.121
**Interventions, n (%)**						
MV	2624 (98.6)	854 (96.9)	0.002	147 (79.0)	68 (72.3)	0.270
RRT	539 (20.3)	32 (3.6)	<0.001	118 (63.4)	23 (24.5)	<0.001
Vasopressor use	2067 (77.7)	547 (62.1)	<0.001	123 (66.1)	46 (48.9)	0.008
**Vital signs**						
Heart rate, bpm	103.0 [90.0, 119.0]	101.0 [88.0, 116.0]	0.007	100.5 [92.0, 120.0]	107.5 [93.0,115.8]	0.523
Respiratory rate, bpm	28.0 [24.0, 32.0]	28.0 [24.0, 32.0]	0.301	25.0 [22.0, 30.0]	25.0 [22.0, 30.0]	0.791
SBP, mmHg	143.0[129.0,159.0]	146.0 [132.0,162.0]	0.033	124.5[102.2,157.0]	108.5 [85.2,138.0]	0.002
DBP, mmHg	78.0 [67.0, 92.0]	79.0 [69.0, 95.0]	0.008	71.0 [60.0, 90.8]	71.0 [59.0, 85.0]	0.371
**Laboratory tests**						
WBC, $*10^9$	12.0 [8.6, 16.7]	11.9 [8.6, 16.6]	0.649	11.0 [8.4, 17.1]	11.4 [8.3, 15.6]	0.823
RBC, $*10^9$	3.5 [3.0, 3.9]	3.4 [3.0, 3.9]	0.658	2.8 [2.5, 3.5]	3.4 [3.0, 3.8]	<0.001
Hemoglobin, g/dL	10.3 [9.0, 11.5]	10.3 [9.0, 11.7]	0.304	8.5 [7.6, 10.2]	10.2 [8.6, 11.4]	<0.001
Hematocrit, %	31.4 [28.0, 35.4]	31.6 [28.0, 35.9]	0.320	26.4 [23.3, 32.1]	31.1 [26.9, 34.3]	<0.001
Potassium, mEq/L	4.4 [4.0, 4.9]	4.3 [3.9, 4.7]	<0.001	4.5 [4.0, 5.2]	4.3 [3.8, 4.9]	0.041
Calcium, mEq/L	8.4 [8.0, 8.8]	8.4 [8.0, 8.9]	0.742	2.0 [1.9, 2.1]	2.0 [1.9, 2.2]	0.042
Anion gap, mmol/L	15.3 [13.0, 18.0]	15.0 [12.0, 17.0]	<0.001	16.6 [13.6, 20.5]	15.3 [13.4, 17.7]	0.007
PaO2, mmHg	73.0 [49.0, 94.0]	75.0 [55.0, 100.0]	0.010	68.5 [42.2, 95.0]	74.5 [50.8, 95.5]	0.159

PaCO2, mmHg	44.0 [38.0, 53.0]	44.0 [38.0, 52.0]	0.322	39.0 [34.0, 48.0]	43.0 [36.2, 48.8]	0.117
pH	7.4 [7.4, 7.4]	7.4 [7.4, 7.4]	0.872	7.4 [7.4, 7.5]	7.4 [7.3, 7.5]	0.546
Glucose, mmol/L	9.3 [7.5, 12.1]	9.1 [7.5, 11.6]	0.137	9.2 [7.0, 11.5]	8.7 [6.8, 11.9]	0.637
BUN on day 1, mmol/L	10.7 [7.1, 16.8]	9.6 [6.1, 13.9]	<0.001	19.2 [12.2, 28.3]	12.3 [8.3,16.5]	<0.001
Creatinine on day 1, umol/L	123.8 [79.6,194.5]	106.1 [70.7,150.3]	<0.001	256.2[171.1,452.4]	146.2 [119.3,187.6]	<0.001
BUN on day 3, mmol/L	11.4 [7.1, 18.2]	10.4 [6.4, 15.7]	<0.001	18.3 [11.2, 27.5]	11.6 [7.4, 17.9]	<0.001
Creatinine on day 3, umol/L	114.9 [70.7,203.4]	97.3 [70.7,150.3]	<0.001	234.7[161.6,393.7]	129.6 [90.4, 175.0]	<0.001
Delta BUN, mmol/L	0.7 [-1.4, 3.2]	0.6 [-1.3, 3.3]	0.392	0.9 [-6.6, 4.8]	-0.4 [-5.1, 2.9]	0.799
Delta creatinine, umol/L	0.0 [-26.5,17.7]	-8.8 [-17.7, 8.8]	0.338	-8.4 [-96.6, 33.0]	-5.2 [-60.9, 13.2]	0.605

CKD, Chronic Kidney Disease; CPD, Chronic Pulmonary Disease; CLD, Chronic Liver Disease; MV, Mechanical Ventilation; RRT, Renal Replacement Therapy; SBP, Systolic Blood Pressure; DBP, Diastolic Blood Pressure; WBC, White Blood Cell counts; RBC, Red Blood Cell counts; BUN, Blood Urea Nitrogen.
