# Supplementary material for: Machine Learning for Predicting Risk and Prognosis of Acute Kidney Disease in Critically Ill Elderly Patients During Hospitalization: Internet-Based and Interpretable Model Study
Source: J Med Internet Res. 2024 May 1;26:e51354. doi: 10.2196/51354 (PMC11097053; doi:10.2196/51354)
Supplement: Multimedia Appendix 4 [file jmir_v26i1e51354_app4.pdf]

Multimedia Appendix 4. The ROC curves in the internal validation of AKD risk prediction models.

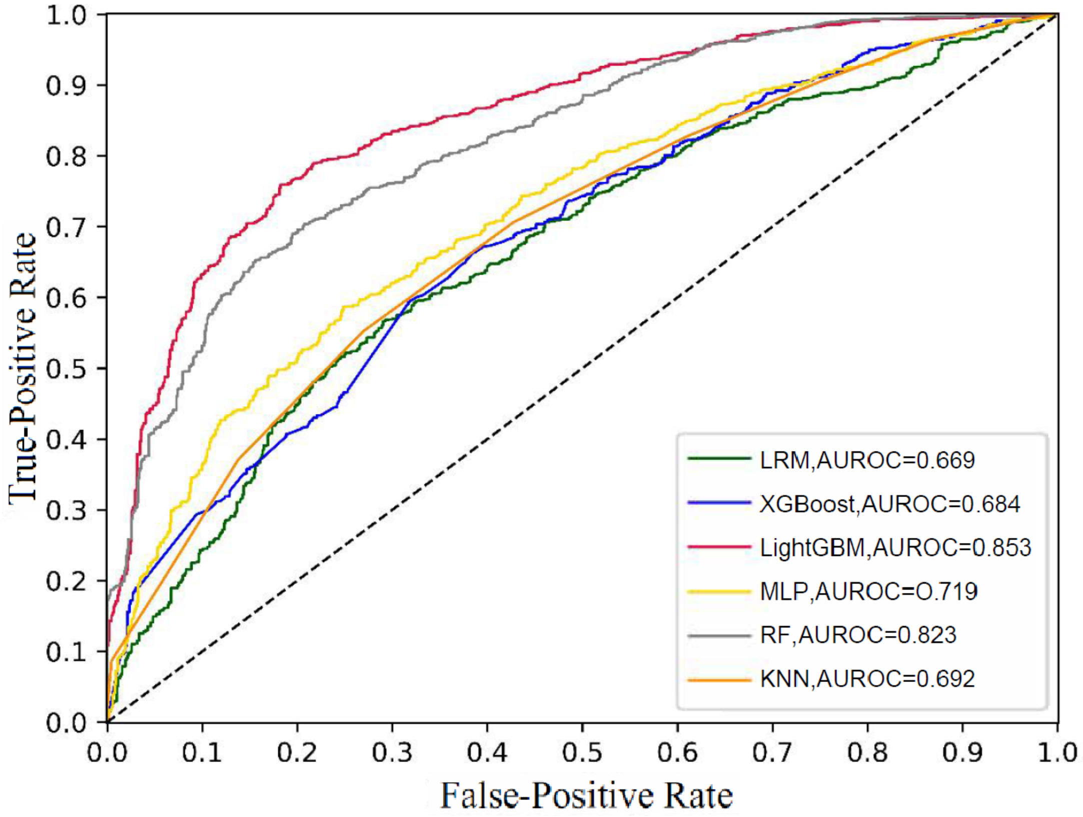

AUROC: area under the receiver operating characteristic curve; DCA: decision curve analysis; KNN: K-nearest neighbor; LightGBM: Light Gradient Boosting Machine; LRM: logistic regression model; MLP: multilayer perceptron; RF: random forest; ROC: receiver operating characteristic; XGBoost: Extreme Gradient Boosting.
