# Supplementary material for: Machine Learning for Predicting Risk and Prognosis of Acute Kidney Disease in Critically Ill Elderly Patients During Hospitalization: Internet-Based and Interpretable Model Study
Source: J Med Internet Res. 2024 May 1;26:e51354. doi: 10.2196/51354 (PMC11097053; doi:10.2196/51354)
Supplement: Multimedia Appendix 6 [file jmir_v26i1e51354_app6.pdf]

Multimedia Appendix 6. The characteristics of elderly patients with AKD.

Variables	MIMIC-IV cohort Death (n=788)	MIMIC-IV cohort Survival (n=1873)	*P*-value	Xiangya hospital cohort AKD (n=77)	Xiangya hospital cohort Non-AKD (n=109)	*P*-value
**Basic information**						
Age, years	76.1 [68.6, 82.7]	72.7 [66.6, 80.0]	<0.001	73.0 [65.0, 80.0]	72.0 [65.0, 78.0]	0.555
Gender, n			0.120			0.129
Male	430 (54.6)	1085 (57.9)		64 (83.1)	79 (72.5)	
Female	358 (45.4)	788 (42.1)		13 (16.9)	30 (27.5)	
Aki stage, n			0.595			0.678
I	596 (75.6)	1448 (77.3)		21 (27.3)	31 (28.4)	
II	178 (22.6)	390 (20.8)		18 (23.4)	27 (24.8)	
III	14 (1.8)	35 (1.9)		38 (49.4)	51 (46.8)	
**Comorbidities, n**						
Sepsis	754 (95.7)	1703 (90.9)	<0.001	26 (33.8)	14 (12.8)	0.001
Hypertension	372 (47.2)	962 (51.4)	0.056	53 (68.8)	81 (74.3)	0.513
Diabetes	291 (36.9)	756 (40.4)	0.107	29 (37.7)	43 (39.4)	0.925
CKD	271 (34.4)	552 (29.5)	0.014	33 (42.9)	53 (48.6)	0.530
CPD	284 (36.0)	679 (36.3)	0.953	16 (20.8)	17 (15.6)	0.474
CLD	98 (12.4)	120 (6.4)	<0.001	20 (26.0)	17 (15.6)	0.119
**Interventions, n**						
MV	778 (98.7)	1846 (98.6)	0.868	73 (94.8)	74 (67.9)	<0.001
RRT	228 (28.9)	311 (16.6)	<0.001	63 (81.8)	55 (50.5)	<0.001
Vasopressor use	682 (86.5)	1385 (73.9)	<0.001	72 (93.5)	51 (46.8)	<0.001
**Vital signs**						
Heart rate, bpm	104.0 [92.0,122.0]	103.0 [90.0,118.0]	0.012	112.0 [94.0, 123.0]	99.0 [89.0,114.0]	0.005
Respiratory rate, bpm	28.0 [25.0, 33.0]	28.0 [24.0, 32.0]	<0.001	26.0 [23.0, 31.0]	25.0 [22.0, 29.0]	0.062
SBP, mmHg	140.0[128.8,156.2]	144.0 [130.0,161.0]	0.001	119.0[103.0,154.0]	131.0 [100.0,159.0]	0.357
DBP, mmHg	76.5 [67.0, 90.0]	78.0 [68.0, 92.0]	0.052	69.0 [60.0, 89.0]	72.0 [61.0, 91.0]	0.454
**Laboratory tests**						
WBC, $*10^9$	12.7 [9.0, 17.8]	11.8 [8.4, 16.3]	0.001	11.9 [9.7, 17.9]	10.5 [8.0, 16.8]	0.102
RBC, $*10^9$	3.4 [2.9, 3.9]	3.5 [3.1, 3.9]	0.001	2.8 [2.6, 3.5]	2.9 [2.5, 3.5]	0.498
Hemoglobin, g/dL	10.0 [8.8, 11.4]	10.3 [9.1, 11.6]	0.001	85.0 [76.0, 100.0]	85.0 [76.0, 103.0]	0.814
Hematocrit, %	30.9 [27.6, 35.2]	31.6 [28.2, 35.5]	0.007	27.4 [23.9, 32.6]	25. [22.7, 31.3]	0.154
Potassium, mEq/L	4.5 [4.0, 4.9]	4.4 [4.0, 4.8]	0.027	4.4 [3.9, 5.1]	4.6 [4.1, 5.2]	0.253
Calcium, mEq/L	8.4 [7.9, 8.8]	8.4 [8.0, 8.8]	0.292	2.0 [1.8, 2.1]	2.0 [1.9, 2.1]	0.271
Anion gap, mmol/L	16.0 [13.0, 20.0]	15.0 [13.0, 18.0]	<0.001	18.5 [15.4, 21.3]	15.4 [12.8, 19.2]	<0.001
PaO2, mmHg	72.0 [46.0, 93.0]	73.0 [51.0, 94.0]	0.095	64.0 [48.0, 95.0]	69.0 [41.0, 95.0]	0.849
PaCO2, mmHg	44.0 [38.0, 53.0]	45.0 [39.0, 53.0]	0.008	39.0 [33.0, 55.0]	39.0 [35.0, 48.0]	0.982

pH	7.4 [7.3, 7.4]	7.4 [7.4, 7.4]	0.018	7.4 [7.4, 7.5]	7.4 [7.3, 7.4]	0.128
Glucose, mmol/L	9.7 [7.6, 12.7]	9.2 [7.4, 11.8]	0.024	9.4 [7.2, 12.0]	8.9 [6.9, 11.3]	0.229
BUN on day 1, mmol/L	12.9 [8.2, 19.7]	10.0 [6.8, 15.4]	<0.001	19.9 [15.2, 32.1]	17.7 [11.2, 24.6]	0.026
Creatinine on day 1, umol/L	141.5 [88.4,212.2]	114.9 [79.6,185.7]	<0.001	244.0[184.3,444.2]	262.8 [167.8,452.4]	0.950
BUN on day 3, mmol/L	13.6 [8.6, 21.8]	10.7 [6.8, 16.8]	<0.001	22.3 [12.9, 29.2]	16.6 [10.8, 25.9]	0.085
Creatinine on day 3, umol/L	137.1 [88.4,238.7]	106.1 [70.7,185.7]	<0.001	250.0[163.2,398.9]	220.0 [159.1,393.7]	0.636
Delta BUN, mmol/L	1.1 [-1.4, 4.3]	0.4 [-1.4, 3.2]	0.006	0.9 [-8.9, 7.2]	0.9 [-5.2, 4.2]	0.700
Delta creatinine, umol/L	0.0 [-17.7,35.4]	-8.8 [-26.5,17.7]	<0.001	-15.2[-109.5, 33.2]	-4.9 [-84.8, 32.4]	0.640

CKD, Chronic Kidney Disease; CPD, Chronic Pulmonary Disease; CLD, Chronic Liver Disease;

MV, Mechanical Ventilation; RRT, Renal Replacement Therapy; SBP, Systolic Blood Pressure;

DBP, Diastolic Blood Pressure; WBC, White Blood Cell counts; RBC, Red Blood Cell counts;

BUN, Blood Urea Nitrogen.
