# Supplementary material for: Machine Learning for Predicting Risk and Prognosis of Acute Kidney Disease in Critically Ill Elderly Patients During Hospitalization: Internet-Based and Interpretable Model Study
Source: J Med Internet Res. 2024 May 1;26:e51354. doi: 10.2196/51354 (PMC11097053; doi:10.2196/51354)
Supplement: Multimedia Appendix 8 [file jmir_v26i1e51354_app8.pdf]

Multimedia Appendix 8. **Internet-based application of LightGBM model for predicting AKD mortality.**

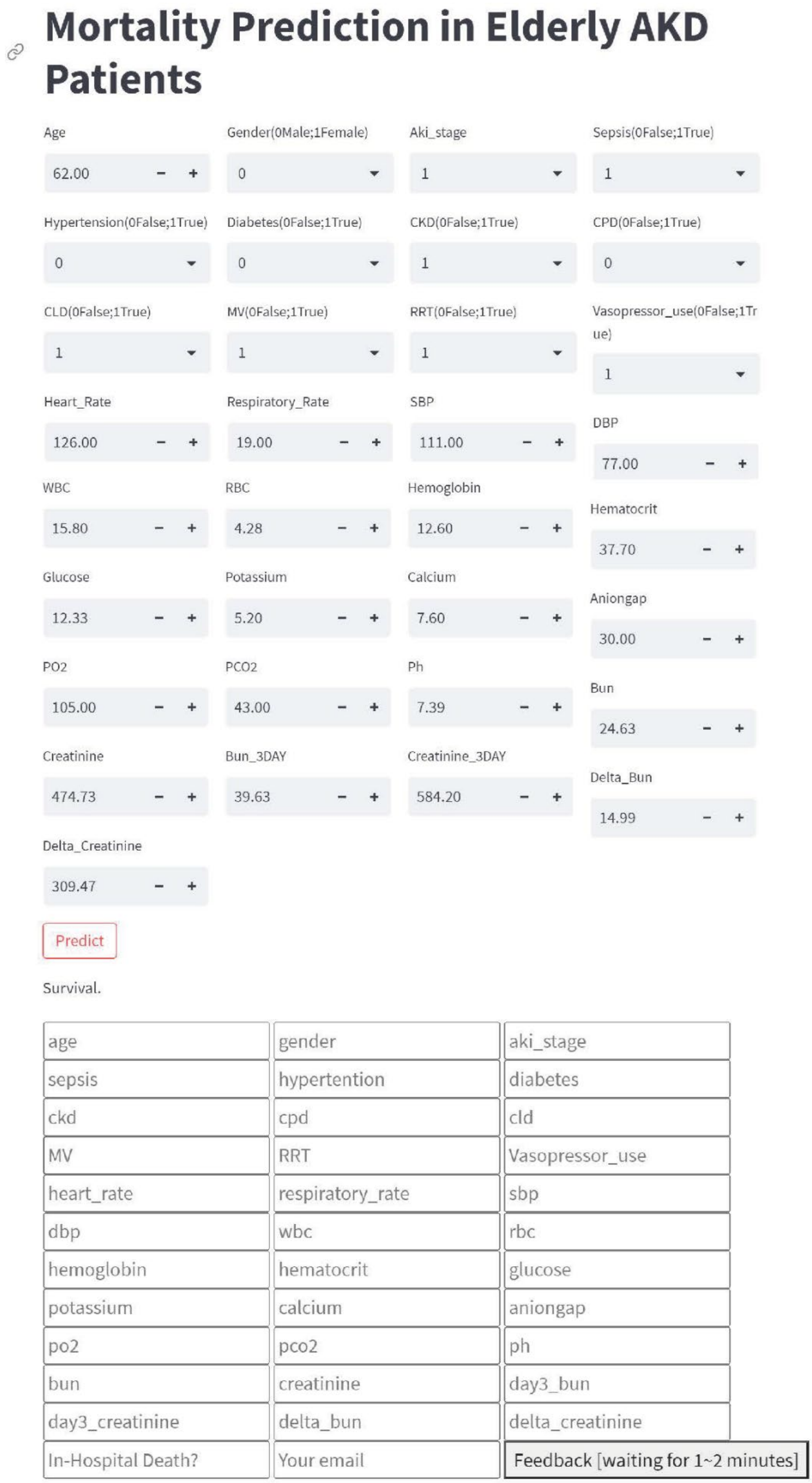

CKD, Chronic Kidney Disease; CPD, Chronic Pulmonary Disease; CLD, Chronic Liver Disease; MV, Mechanical Ventilation; RRT, Renal Replacement Therapy; SBP, Systolic Blood Pressure; DBP, Diastolic Blood Pressure; WBC, White Blood Cell counts; RBC, Red Blood Cell counts; BUN, Blood Urea Nitrogen.
